# PAK1 inhibition synergistically enhances the anti-tumor efficacy of PARP inhibitors in ovarian cancers

**DOI:** 10.1016/j.gendis.2025.101887

**Published:** 2025-10-21

**Authors:** Changying Li, Xinyan Li, Ming Gao, Min Deng, Ye-Xiong Li, Zhenkun Lou

**Affiliations:** a State Key Laboratory of Molecular Oncology and Department of Radiation Oncology, National Cancer Center/National Clinical Research Center for Cancer/Cancer Hospital, Chinese Academy of Medical Sciences and Peking Union Medical College, Beijing 100021, China; b State Key Laboratory of Environmental Chemistry and Ecotoxicology, Research Center for Eco-Environmental Sciences, Chinese Academy of Sciences, Beijing 100085, China; c Department of Oncology, Mayo Clinic, Rochester, MN 55905, USA

**Keywords:** HR repair, Olaparib, Ovarian cancer, PAK1, Synthetic lethality

## Abstract

Poly (ADP-ribose) polymerase inhibitors (PARPi) demonstrate effective treatment outcomes in ovarian cancer patients with BRCA1/2 mutations or homologous recombination (HR) repair deficiencies, leveraging the principle of synthetic lethality. However, PARPi resistance and HR proficiency remain significant challenges in the clinical management of PARPi, necessitating the development of novel strategies for PARPi therapy in ovarian cancer. Our previous research identified PAK1's involvement in replication stress-induced cytotoxicity. Nonetheless, whether PAK1 also affects HR repair and PARPi sensitivity in ovarian cancer remains unresolved. In this research, we found that the expression of PAK1 correlated with an unfavorable prognosis in ovarian cancer. Depletion of PAK1, introduction of a kinase-dead mutation, or treatment with the inhibitor IPA-3 could reduce HR repair efficiency and increase ovarian cancer cell sensitivity to the PARP inhibitor olaparib. The combination of olaparib and IPA-3 synergistically increased olaparib-induced DNA replication stress and double-stranded breaks. Using cell line-derived xenograft, patient-derived organoid, and patient-derived xenograft models, we discovered that IPA-3 potentiated the therapeutic efficacy of olaparib both *in vivo* and *ex vivo*. Collectively, our findings suggest that targeting PAK1 might offer a new avenue for increasing the sensitivity of olaparib and improving the outcomes of ovarian cancer patients.

## Introduction

Ovarian cancer (OC) ranks as the most lethal gynecological malignancy, with many cases diagnosed at an advanced stage after metastasis beyond the ovaries.^1^, ^2^, ^3^ Platinum-based chemotherapy combined with surgery is the primary treatment for newly diagnosed advanced OC; however, most patients experience recurrence, and treatment efficacy diminishes over time.^4^^,^^5^ Therefore, developing novel treatment approaches is essential to enhance outcomes for OC patients. Poly (ADP-ribose) polymerase (PARP) inhibitors have recently emerged as a revolutionary treatment option for OC patients.^6^^,^^7^ PARP1, PARP2, and PARP3 enzymes play a pivotal role in DNA single-strand break repair and cellular response to replication stress.^8^, ^9^, ^10^ PARP inhibition leads to the accumulation of single-strand breaks, stalling of replication forks, and ultimately conversion to double-strand breaks (DSBs).^11^^,^^12^ DSBs can be repaired through homologous recombination (HR) and non-homologous end-joining (NHEJ). In case they are not repaired properly, it will result in genome instability and even cause cell death.^13^ Therefore, PARP inhibitors have exhibited significant clinical benefit for patients who have HR gene deficiency (HRD), via the remarkable mechanism of “synthetic lethality”.^14^^,^^15^ Approximately 50% of OC patients exhibit HRD, with around 22% carrying germline or somatic mutations in BRCA1/2.^16^^,^^17^ Olaparib, the first PARP inhibitor approved by regulatory authorities for treating metastatic OC, is particularly effective in HRD-positive cases.^18^^,^^19^ However, similar to other targeted therapies, PARP inhibitor resistance also occurred due to the recovery of HR repair, the change of the drug target, etc.^20^^,^^21^ Therefore, combining PARP inhibitors with inhibitors of HR repair genes represents a promising strategy to enhance PARP inhibitor efficacy in both BRCA-proficient tumors and OC patients with PARP inhibitor resistance.

P21-activated kinase 1 (PAK1), a serine–threonine kinase, acts as an effector for small GTPases like Rac1 and Cdc42, and is crucial for cellular functions such as motility, apoptosis, proliferation, and cell cycle regulation.^22^^,^^23^ PAK1 dysregulation disrupts cellular homeostasis and is linked to diseases like heart disease, neurological disorders, and cancers.^24^^,^^25^ Upon binding to CDC42 and RAC1, PAK1 experiences a conformational shift and separation of its homodimer, thus promoting the phosphorylation of its activation loop.^26^^,^^27^ Amplification of the PAK1 gene, which leads to elevated PAK1 protein levels, or hyperactivation of PAK1 due to mutations of its upstream regulators, has been observed in several cancer types and is often associated with aggressive tumor behaviors, including enhanced invasiveness, occurrence of metastasis, and greater resistance to chemotherapy.^28^^,^^29^ Therefore, modulating PAK1 activity holds promise as a therapeutic avenue worth exploring in certain cancers. Several compounds targeting PAK1 have been developed as preclinical agents for advanced solid tumors and lymphomas in recent years.^30^^,^^31^ However, the combination therapies of PAK1 inhibitors with other agents in OC are still rarely reported and deserve further investigation.

Our previous study showed that PAK1 was involved in replication stress response by promoting the recruitment of replication protein A1 (RPA1) onto stalled DNA replication forks.^32^ Given that the RPA complex serves as a platform for recruiting key HR factors to repair DSBs,^33^^,^^34^ we further investigated the role of PAK1 in HR repair in this study. As a result, we found that PAK1 was a new regulator of HR repair through its kinase activity, but did not affect NHEJ repair. Inhibition of PAK1 by its inhibitor IPA-3 sensitized OC cells to olaparib by inducing more replication stress and DSB damage. Additionally, using cell line-derived xenograft (CDX), patient-derived organoids (PDO), and patient-derived xenograft (PDX) models, we revealed that the combination of olaparib with IPA-3 synergistically suppressed tumor growth *in vivo* and *ex vivo*. These findings indicate that a synergistic approach targeting both PAK1 and olaparib could be an effective therapeutic strategy for OC.

## Materials and methods

### Cell culture

The HEK293T, Ovcar8, and SKOV-3 cell lines were obtained from the Cell Resource Center of the Institute of Basic Medical Sciences (China) and cultured in Dulbecco's Modified Eagle Medium supplemented with 10% fetal bovine serum at 37 °C in a 5% CO_2_ atmosphere.

### Plasmids, reagents, and antibodies

The FLAG-PAK1 K299R mutant was created through site-directed mutagenesis (NEB). Chlorodeoxyuridine (Cidu), 5-iodo-2′-deoxyuridine (Idu), and the PAK1 inhibitor IPA-3 were sourced from MedChemExpress, while the PARP inhibitor olaparib was acquired from TargetMol. Anti-RPA1 (A300–241A, 1:5000) was procured from Bethyl Laboratories. PAK1, PAK2, PAK3 shRNA sequence were shown in Table 1. Antibodies were sourced as follows: anti-FLAG (F1804, 1:2000) was obtained from Sigma; anti-RPA32 (sc-56770, 1:2000) was obtained from Santa Cruz; anti-pS345 Chk1 (2348, 1:1000), anti-phospho-PAK1 (Thr423) (2601, 1:1000), anti-cleaved caspase-3 (9664, 1:1000), and anti-histone H3 (4499, 1:2000) were obtained from CST Signaling; anti-GAPDH (60004-1-lg, 1:2000), anti-PAK1 (21401-1-AP, 1:1000), anti-PAK2 antibody (19979-1-AP, 1:2000), and anti-PAK3 antibody (32848-1-AP, 1:2000) were obtained from Proteintech Group; anti-RAD51 antibody (GTX100469, dilution 1:1000) was obtained from Genetex.

### NHEJ and HR assay

Stable cell lines were established through lentiviral transduction following standard protocols. For transient DNA repair assays, HEK293T cells were co-transfected with 0.2 μg of DR-GFP (homology-directed repair reporter), 0.2 μg of EJ5-GFP (non-homologous end joining reporter), 0.2 μg of pCBA-I-SceI (meganuclease expression vector), and 0.1 μg of mCherry (transfection efficiency control) using polyethylenimine. Cells were harvested 48 h post-transfection and analyzed with a BD FACSAria III flow cytometer (Attune NxT), followed by data processing using FlowJo v10.8 software. GFP-positive populations (a marker of successful DNA repair) were quantified solely within mCherry-positive cells (the transfected population) to normalize transfection efficiency.

### Colony formation assay

To evaluate clonogenic survival, Ovcar8 or SKOV-3 cells were seeded in 6-well plates at a density of 500–1000 cells/well. Following a 24-h attachment period, cells were subjected to experimental interventions as required. Subsequently, cells were maintained in complete medium at 37 °C for 10–14 days to allow for colony formation. Colonies were fixed with 4% methanol for 15 min, stained with 0.5% crystal violet for 30 min, and manually quantified using a light microscope. The colony counts were then adjusted for plating efficiencies.

### Western blotting

Cells were harvested and lysed in the nuclear and cytoplasmic extraction (NETN) buffer for 30 min,^35^ followed by centrifugation at 12,000 *g* for 15 min. The supernatant containing the proteins was then combined with the loading buffer and subjected to Western blotting analysis using standard protocols.

### Co-immunoprecipitation assay

Cells were lysed in NP-40 buffer containing protease and phosphatase inhibitors.^36^ Protein was incubated overnight at 4 °C with specific antibodies, such as anti-PAK1 or anti-CHK1. Protein A/G agarose beads (Santa Cruz Biotechnology) were then added, and the mixture was incubated at 4 °C for 2 h. After three washes with lysis buffer, bound proteins were eluted by heating in SDS sample buffer.

### Chromatin fractionation

Ovcar8 cells were collected and incubated in low-salt buffer on ice for 30 min. After centrifugation, the supernatant was saved as the soluble fraction. The pellet was resuspended in 0.2 N HCl and kept on ice for 20 min, followed by sonication and centrifugation. The supernatant, containing chromatin-associated proteins, was neutralized with 1 M Tris–HCl (pH 8.0) and analyzed by Western blotting.

### Immunofluorescence staining

Ovcar8 and SKOV-3 cells were seeded on glass coverslips and treated as per the experimental design. At the endpoint, cells were fixed with 4% paraformaldehyde at room temperature for 15 min and then permeabilized with 0.1% Triton X-100 for 10 min. Non-specific binding was blocked by incubating cells with 5% goat serum for 30 min, followed by overnight incubation with primary antibodies at 4 °C. After washing, secondary antibodies were incubated at room temperature for 1 h, and nuclei were stained with DAPI. Coverslips were mounted on glass slides with an anti-fade reagent and imaged using a Nikon ECLIPSE E800 fluorescence microscope. Fluorescence intensity was quantified using ImageJ software.

### Immunohistochemistry analysis

Tumor tissues were fixed in 10% formalin, embedded in paraffin, and sectioned into 4 μm thick slices. Antigen retrieval was carried out by microwaving the tissue sections in citrate buffer (pH 6.0). Endogenous peroxidase activity was quenched by incubating the sections with 3% hydrogen peroxide for 10 min. The sections were then incubated at 4 °C overnight with primary antibodies targeting Ki-67 (1:200), γ-H2AX (1:500), and cleaved caspase-3 (1:300). Following primary antibody incubation, horseradish peroxidase-conjugated secondary antibodies were applied, and immunoreactivity was visualized. Nuclei were counterstained with hematoxylin. Ki-67, γ-H2AX, and cleaved caspase-3 expression were assessed by counting positive cells in at least five random fields per section.

### DNA fiber assay

Ovcar8 cells were initially labeled with 25 μM IdU for 30 min, followed by a 4-h exposure to IPA-3, olaparib, or their combination. Subsequently, 200 μM CldU was added for an additional 30 min before harvesting. Cells were re-suspended in a lysis buffer containing 200 mM Tris–HCl (pH 7.4), 50 mM EDTA, and 0.5% SDS for 10 min, and then gently flowed down a slide inclined at 15 °. The slides underwent fixation, neutralization, blocking, incubation with primary and secondary antibodies, and visualization, as previously described.

### Isolation of proteins on nascent DNA

Ovcar8 cells were labeled with 10 μM EdU for 20 min, followed by a 4-h treatment with IPA-3, olaparib, or their combination. The cells were fixed at room temperature and then neutralized with 1.25 M glycine. After permeabilization, cells were incubated with click reaction buffer, resuspended in lysis buffer, and sonicated. The samples were then incubated with streptavidin and subjected to Western blotting analysis according to standard protocols.

### Fluorescent reporter system for DSB repair assay

A fluorescent reporter system was utilized to examine PAK1's role in repairing DNA DSBs, focusing on the HR and NHEJ pathways. Cells were cultured in 6-well plates and transfected with repair reporter plasmids using Lipofectamine 2000 reagent and exposed to 1 μM etoposide or 10 Gy ionizing radiation to induce DSBs. After 24–48 h of incubation, GFP fluorescence, indicative of HR or NHEJ activity, was used to assess DSB repair. GFP-positive cells were quantified via fluorescence microscopy or flow cytometry, and repair efficiency was analyzed with ImageJ software.

### Cell cycle analysis and Edu analysis

To assess the impact of PAK1 depletion on cell cycle progression, flow cytometry was conducted. Cells were collected and fixed in 70% ethanol overnight at −20 °C. After fixation, they were washed with phosphate-buffered saline, treated with 100 μg/mL RNase A at 37 °C for 30 min, and stained with 50 μg/mL propidium iodide in the dark for 30 min. The cell cycle distribution was then analyzed using a BD FACSCalibur flow cytometer, and the percentage of cells in each phase (G1, S, and G2/M) was determined using FlowJo software. EdU assay was performed using an EdU assay kit (Yeasen, China).

### Tumor xenograft

Experiments were conducted in accordance with the ethical guidelines approved by Cancer Hospital Chinese Academy of Medical Sciences. A 100 μL mixture containing 2 × 10^6^ OVCAR8 or SKOV-3 cells, mixed with 50% Matrigel (BD Biosciences), or ovarian PDXs from Nanchang Royo Biotech Co., Ltd., China, was subcutaneously injected into the flanks of 6- to 8-week-old female athymic nude BALB/C mice. Upon reaching approximately 50 mm^3^ in tumor volume, mice were assigned to one of four groups: control (phosphate-buffered saline), IPA-3 (50 mg/kg), olaparib (50 mg/kg), or the combination (IPA-3 and olaparib). Treatments were administered intraperitoneally three times a week. Tumor volume was measured using calipers and calculated with the formula 0.5 × length × width^2^. Mice were euthanized at specified time points post-treatment, and tumor tissues were immediately fixed in formalin for subsequent immunohistochemical staining (hematoxylin-eosin, Ki-67, cleaved caspase-3, and γ-H2AX). Statistical analysis was performed using Student's *t*-test. All procedures involving animals adhered to the ethical standards set by the Animal Ethics Committee of the National Cancer Center/National Clinical Research Center for Cancer/Cancer Hospital, Chinese Academy of Medical Sciences, and Peking Union Medical College. Written informed consent was obtained from all patients, and the study protocol was approved by the Human Institutional Review Board of Nanchang Royo Biotech Co., Ltd., China (Permit No. 2021012). Serum alanine aminotransferase (ALT) and aspartate transaminase (AST) levels were measured using ALT and AST activity assay kits (Nanjing JianCheng Bioengineering Institute, Jiangsu, China) following the manufacturer's instructions.

### Primary patient-derived organoid culture and treatment

Two OC PDOs were established from fresh tumor biopsies and cultured in Matrigel with organoid growth medium (Lonza) supplemented with epidermal growth factor (EGF), R-spondin, Noggin, and B27. Organoids were passaged every 7–10 days by mechanical disruption. For drug treatments, PDOs were incubated with olaparib (1 μM) and IPA-3 (10 μM), either alone or in combination, for 48–72 h. Organoid morphology, including formation and diameter, was assessed using a bright-field microscope. Caspase-3 cleavage was evaluated by immunofluorescence staining. The medical ethics committee of the Second Affiliated Hospital of Nanchang University approved the study protocol (Permit No. 2021012).

### Patient-derived xenograft model

PDX models were created by subcutaneously implanting fresh ovarian tumor biopsies into 6-to-8-week-old female athymic nude mice (Nu/Nu). Tumor growth was consistently tracked using caliper measurements, and the volume was determined with the formula: volume = (length × width^2^)/2. Mice with tumors of approximately 100 mm^3^ were randomly allocated to treatment groups: vehicle, olaparib (10 mg/kg), IPA-3 (20 mg/kg), or the combination (olaparib and IPA-3). Treatments were given via oral gavage on alternate days for three weeks. Tumor growth inhibition was assessed by monitoring changes in tumor volume and weight. Upon treatment completion, tumors were excised and analyzed for Ki67, γ-H_2_AX, and cleaved caspase-3 levels using immunohistochemistry.

### RNA sequencing and quantitative real-time PCR

Total RNA was isolated from cells treated with IPA-3, olaparib, or their combination using the RNeasy Mini Kit (Qiagen), followed by mRNA purification with poly-T oligo-attached beads. The integrity of the RNA was evaluated using the Agilent 2100 Bioanalyzer. cDNA libraries were prepared with the TruSeq RNA Library Prep Kit and sequenced on the Illumina NovaSeq platform, generating 150-bp paired-end reads. Gene expression data were quantified using FeatureCounts, and differential expression analysis was conducted with DESeq2, using an adjusted *P*-value threshold of 0.05. Gene Ontology (GO) and Kyoto Encyclopedia of Genes and Genomes (KEGG) pathway enrichment analysis of differentially expressed genes was performed using clusterProfiler. The RNA sequencing data are accessible in the GEO repository. Gene Set Enrichment Analysis (GSEA) was applied to identify related expression patterns. Total RNA was extracted, reverse-transcribed into cDNA, and then subjected to quantitative real-time PCR for quantification. The primer sequences are listed in Table 1.

**Table 1:** ShRNA targeting sequence and PCR primer sequences.

Name	Sequence
PAK1 shRNA-1	CTTCTCCCATTTCCTGATCTA
PAK1 shRNA-2	CCAAGAAAGAGCTGATTATTA
PAK2 shRNA-1	CCGGCGGGATTTCTTAAATCGATGTCTCGAGACATCGATTTAAGAAATCCCGTTTTTTG
PAK2 shRNA-2	GATCCCCCTGCATAACCTGAATGAAATTCAAGAGATTTCATTCAGGTTATGCAGTTTTTA
PAK3 shRNA-1	TAGCAGCACATCAGTCGAATA
PAK3 shRNA-2	CCCAATATTGTCAATTATTTA
GAPDH-F	CTGGGCTACACTGAGCACC
GAPDH-R	AAGTGGTCGTTGAGGGCAATG
APBB1-F	GGAGGGGACGTTGACCTTC
APBB1-R	TTTTGTGGTAAGAGAGCTGACG
NPAS2-F	ACACCCTTTCAAGACCTTGCC
NPAS2-R	AGGTTCGTCAACTATGCACATTT
LRIF1-F	AGGATTTGAGAGTGTGCCTTACT
LRIF1-R	ACCAAACTGCTAAAGGAATCGAA
SFRP1-F	ACGTGGGCTACAAGAAGATGG
SFRP1-R	CAGCGACACGGGTAGATGG
HMGA2-F	ACCCAGGGGAAGACCCAAA
HMGA2-R	CCTCTTGGCCGTTTTTCTCCA
EYA4-F	AGACTCAGTATTCGGGGATGC
EYA4-R	CCCAAATCGTAAGTGGGCAAG
EYA1-F	GGACTATCCGTCTTATCCCAGT
EYA1-R	GCTGCTGGTCATATAATGTGCTG
HAVCR1-F	GCGTATATTGTTGCCGTGTTG
HAVCR1-R	TGACGGTTGGAACAGTTGTGA

### Bioinformatics analysis

The cBioPortal for Cancer Genomics (http://www.cbioportal.org, version v3.2.11) is an online tool that integrates and visualizes data from major cancer research initiatives, including The Cancer Genome Atlas (TCGA) and the International Cancer Genome Consortium (ICGC). This platform provides gene-level information along with clinical outcomes such as overall survival, progression-free survival, disease-free survival, and disease-specific survival. In this study, cBioPortal was utilized to explore and visualize genetic alterations in HR-related genes in OC. The prognostic impact of PAK1 expression was assessed using the Kaplan–Meier plotter. Patients were categorized into high and low PAK1 expression groups based on the median expression value, and their survival data were compared.

### Statistical analysis

Data presented in bar and line graphs were shown as mean ± standard error of the mean based on three independent experiments. Statistical significance was determined using Student's *t*-test. The following markers indicate significance levels: ∗*P* < 0.05, ∗∗*P* < 0.01, and ∗∗∗*P* < 0.001.

## Results

### PAK1 expression is adversely associated with the overall survival of OC patients

**Figure 1:**
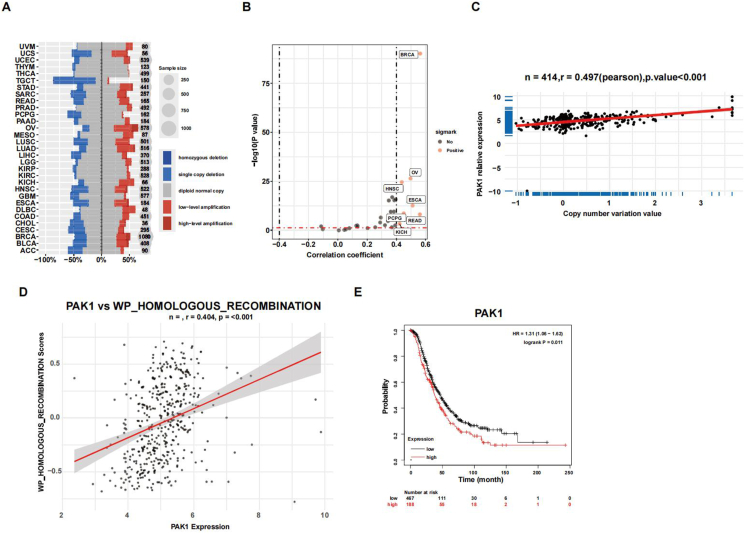
**PAK1 expression is adversely associated with the overall survival of ovarian cancer patients.(A)** Genetic alterations of PAK1 across different cancer types, as retrieved from the cBioPortal database. **(B)** PAK1 expression patterns across a variety of cancers. **(C)** Association between PAK1 expression levels and copy number variations. **(D)** Correlation of PAK1 expression with homologous recombination (HR) status. **(E)** Kaplan–Meier survival analysis comparing ovarian cancer patient outcomes with low (*n* = 467) and high (*n* = 188) PAK1 expression levels.

To investigate whether PAK1 was associated with OC and DNA damage repair, we conducted an analysis using the cBioPortal database. Our findings revealed the pan-cancer prevalence of amplification in PAK1 and frequency mutation of HR-related genes, especially OC (Fig. 1A). The expression of PAK1 in a variety of cancers was significantly increased (Fig. 1B) and PAK1 expression was positively correlated with copy number variation (Fig. 1C). Moreover, PAK1 expression was strongly correlated with HR (Fig. 1D). Kaplan–Meier survival curves showed that OC patients with elevated PAK1 expression had lower overall survival than those with reduced PAK1 expression (Fig. 1E). These findings suggest that PAK1 might be a viable target for treating OC.

### PAK1 depletion inhibits HR repair and sensitizes OC cells to olaparib

**Figure 2:**
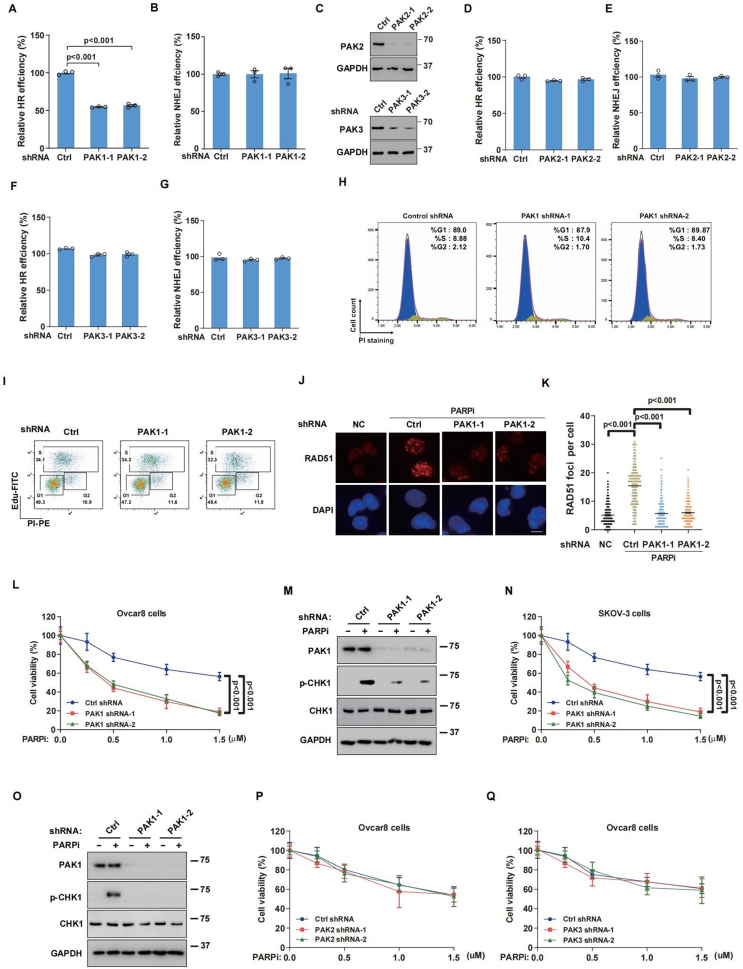
**PAK1 regulates homologous recombination (HR) repair and olaparib sensitivity in ovarian cancer cells.(A, B)** HR (A) and non-homologous end-joining (NHEJ) (B) repair efficiencies in control and PAK1-depleted HEK293T cells, evaluated using HR and NHEJ reporter systems. Data were presented as mean ± standard error of the mean from three independent experiments. **(C)** Western blotting analysis of PAK2 and PAK3 in control and PAK2/PAK3-depleted HEK293T cells. **(D**–**G)** HR (D, F) and NHEJ (E, G) repair efficiencies in control and PAK2/PAK3-depleted HEK293T cells, evaluated using HR and NHEJ reporter systems. **(H, I)** Cell cycle distribution in control and PAK1-depleted Ovcar8 cells, analyzed by flow cytometry. **(J, K)** RAD51 foci formation in Ovcar8 cells treated with 10 μM olaparib for 24 h: (J) representative images and (K) quantification. More than 200 cells were analyzed per experiment. **(L, N)** The survival of control or PAK1-depleted Ovcar8 (L) and SKOV-3 (N) cells, assessed by colony formation assay. **(M, O)** Phosphorylation of CHK1 in control or PAK1-depleted Ovcar8 (M) and SKOV-3 (O) cells, treated with 10 μM olaparib for 6 h. **(P, Q)** The survival of control, (P) PAK2-depleted, or (Q) PAK3-depleted Ovcar8 cells, assessed by colony formation assay. Error bars represent the standard error of the mean from three independent experiments. Scale bars = 50 μm.

We employed a fluorescent reporter system to investigate PAK1's regulatory role in HR and NHEJ repair during DSBs. PAK1 depletion resulted in a marked reduction in HR efficiency compared with control cells (Fig. 2A), whereas PAK1 did not influence NHEJ repair efficiency (Fig. 2B). Moreover, we found that PAK2 and PAK3 did not affect NHEJ and HR repair processes (Fig. 2C–G). In addition, no significant difference in cell cycle distribution was detected between the control and PAK1-depleted cells (Fig. 2H and I), indicating that PAK1-mediated HR repair is not due to cell cycle arrest. Moreover, PARP inhibitors are known to induce cytotoxic effects by causing SSBs to convert to or replication fork collapse into DSBs, which could be subsequently repaired by RAD51-mediated HR. Figure 2J and K demonstrated that depleting PAK1 significantly decreased RAD51 foci formation after olaparib treatment, reinforcing PAK1's role in HR-based DNA repair.

PARP inhibitors trigger synthetic lethality in OC tumors with HR deficiency^37^; thus, we hypothesized that PAK1 depletion-induced HR defect might sensitize cells to olaparib. As shown in Figure 2L and N, PAK1 depletion significantly increased the sensitivity of olaparib in Ovcar8 and SKOV-3 OC cell lines, but not PAK2 and PAK3 (Fig. 2P and Q). It is reported that the ATR/CHK1 pathway activation protects the genome against DNA damage and replication stress upon PARP inhibitor treatment.^38^^,^^39^ Our results also showed that knocking down PAK1 markedly decreased olaparib-induced CHK1 phosphorylation (Fig. 2M–O). Taken together, these results demonstrate that PAK1 is critical for HR repair and olaparib sensitivity.

### PAK1 regulates HR repair and olaparib sensitivity, dependent on its kinase activity

**Figure 3:**
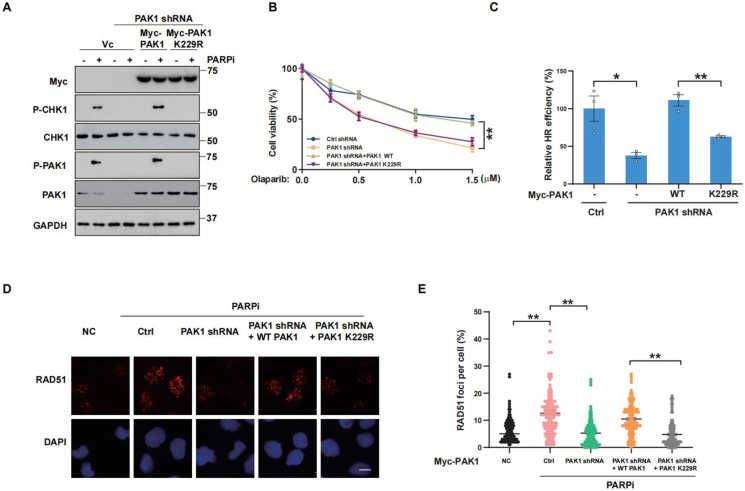
**PAK1 regulates homologous recombination (HR) repair and olaparib sensitivity dependent on its kinase activity. PAK1-depleted cells were transfected with wild-type PAK1 or the K299R kinase mutant for 24 h.(A)** Western blotting analysis of PAK1 and CHK1 phosphorylation in transfected Ovcar8 cells treated with 10 μM olaparib for 6 h. **(B)** The survival of transfected Ovcar8 cells treated with different concentrations of olaparib for 2 weeks, assessed by colony formation assay. **(C)** HR activity in transfected HEK293T cells co-transfected with HR reporter plasmids, followed by HR assay after 48 h. **(D, E)** RAD51 foci formation in transfected Ovcar8 cells treated with 10 μM olaparib for 24 h: (D) representative images and (E) quantification. Over 200 cells were analyzed in each experiment. Error bars represent the standard error of the mean from three independent experiments.

We next probed into whether the serine/threonine kinase activity of PAK1 was essential for HR repair. We evaluated DNA repair efficiency by reconstructing either the wild-type PAK1 or its catalytically inactive mutant (K229R). As depicted in Figure 3A, K229R PAK1 decreased the CHK1 phosphorylation induced by olaparib, whereas wild-type PAK1 notably enhanced CHK1 phosphorylation, suggesting that PAK1 modulates DNA repair relying on its catalytic activity. Moreover, K229R PAK1 substantially augmented olaparib sensitivity (Fig. 3B). Figure 3C showed that HR efficiency was significantly reduced in K229R PAK1 cells compared with wild-type PAK1 cells. In addition, K229R PAK1 reduced the formation of RAD51 foci (Fig. 3D and E). These findings demonstrate that PAK1 facilitates RAD51 recruitment and HR repair through its serine/threonine kinase activity.

### Inhibition of PAK1 by IPA-3 attenuates HR repair and enhances olaparib sensitivity

**Figure 4:**
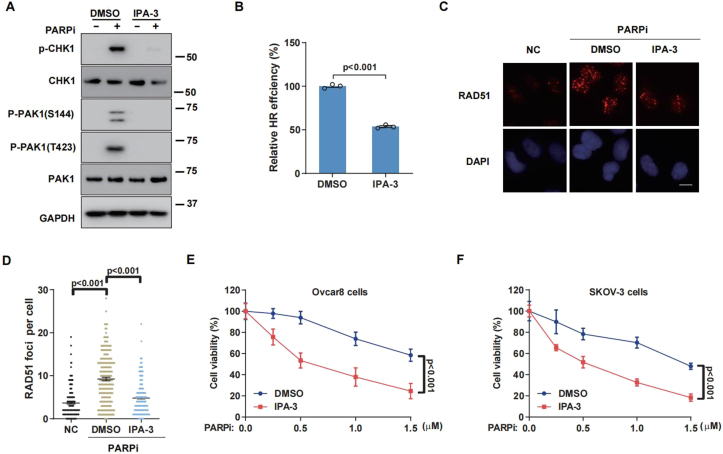
**PAK1 inhibition enhances the efficiency of olaparib in ovarian cancer cells.(A)** Western blotting analysis of PAK1 and CHK1 phosphorylation in Ovcar8 cells treated with 10 μM olaparib, 10 μM IPA-3, or their combination for 6 h. **(B)** Homologous recombination (HR) efficiency in HEK293T cells transfected with HR reporter plasmids, treated with olaparib, IPA-3, or both, followed by HR assay. **(C, D)** RAD51 foci formation in Ovcar8 cells treated with olaparib, IPA-3, or their combination for 24 h: (C) representative images and (D) quantification. More than 200 cells were analyzed per experiment. **(E, F)** The survival of Ovcar8 (E) and SKOV-3 (F) cells treated with olaparib alone or in combination with IPA-3, assessed by colony formation assay. Error bars represent the standard error of the mean from three independent experiments. Statistical significance was determined by a two-tailed *t*-test, with *P*-values < 0.05 considered significant.

IPA-3 is an allosteric inhibitor of PAK1 kinase activity, capable of suppressing cell proliferation and tumor growth both *in vitro* and *in vivo*.^40^^,^^41^ Therefore, we used IPA-3 to further validate whether PAK1 inhibition could regulate HR repair. Figure 4A–D demonstrated that inhibiting PAK1 phosphorylation and activation with IPA-3 effectively reduced HR repair efficiency and RAD51 foci formation. In addition, inhibition of PAK1 sensitized the two OC cell lines to olaparib (Fig. 4E and F). The combination of olaparib and IPA-3 reduced CHK1 activation more effectively than olaparib alone, confirming their synthetic lethality effect on OC cells (Fig. 4A).

### Olaparib and IPA-3 combination induces more replication stress and DNA damage

**Figure 5:**
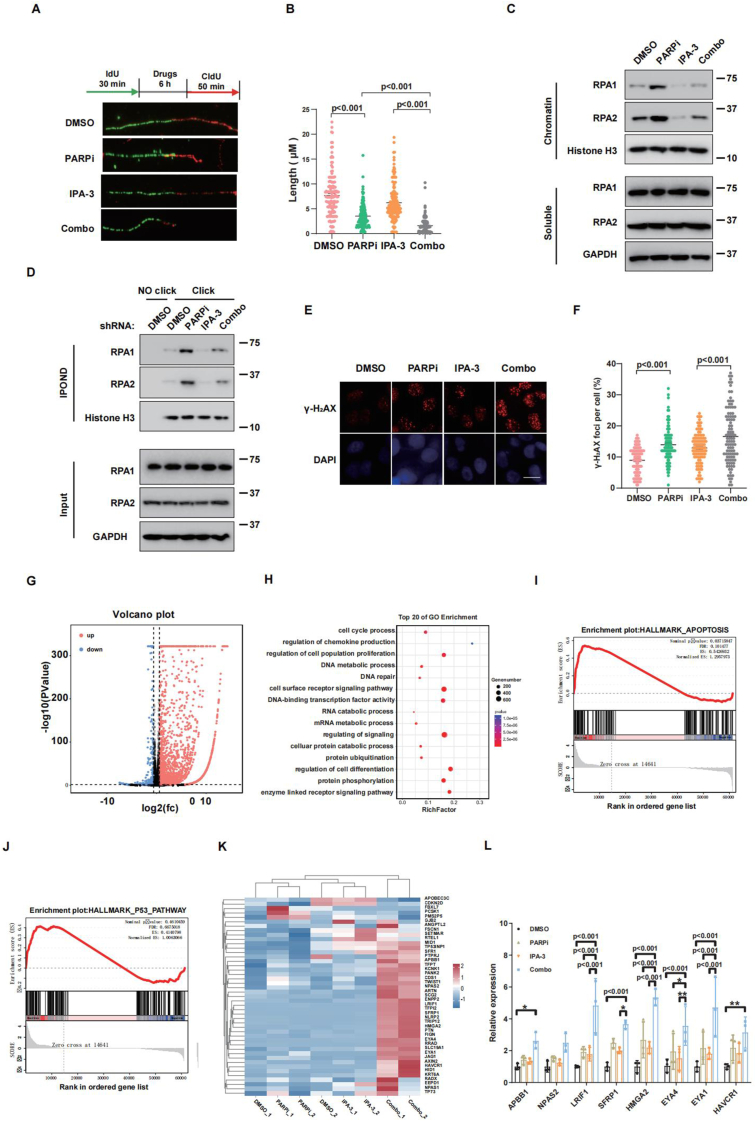
**PAK1 inhibition promotes olaparib-induced replication stress and DNA damage.(A, B)** DNA fiber assay for the length of CIdU (red) tracks in Ovcar8 cells treated with olaparib, IPA-3, or their combination for 6 h: (A) representative images and (B) quantification. Data were expressed as mean ± standard deviation, analyzed by a two-tailed unpaired *t*-test. **(C, D)** Immunoblot analysis of chromatin and soluble fractions of Ovcar8 cells treated with olaparib, IPA-3, or both for 6 h, probing for the indicated antibodies (C), or IPOND (isolation of proteins on nascent DNA) analysis of RPA1 and RPA2 at replication forks (D). **(E, F)** γ-H2AX foci formation in Ovcar8 cells treated with olaparib, IPA-3, or their combination for 24 h: (E) representative images and (F) quantification. More than 100 cells were counted per experiment. **(G)** RNA sequencing analysis of Ovcar8 cells treated with olaparib and IPA-3. Differentially expressed genes were classified based on fold change ≥ 1.5 or ≤ 0.5, with *P* < 0.05. **(H)** Biological process analysis of up- and down-regulated genes in the combination treatment compared with IPA-3 alone. **(I, J)** GSEA of up- and down-regulated genes in the combination treatment compared with IPA-3 alone. **(K)** The heatmap displaying up-regulated genes associated with DNA repair. **(L)** The quantitative real-time PCR showed the up-regulated genes associated with DNA repair. Error bars represent the standard error of the mean from three independent experiments. Statistical significance was determined by a two-tailed *t*-test. Two-sided *P*-values < 0.05 were considered significant. Scale bars = 50 μm.

Our previous data showed that PAK1 directly phosphorylated and stabilized RPA1 on the stalled replication fork to maintain genome stability.^32^ Therefore, it is plausible that the combined effect of olaparib and IPA-3 on cell death is partially attributed to reduced RPA recruitment at stalled replication forks. In this study, we initially examined the impact of IPA-3 on replication stress induced by olaparib. Figure 5A and B showed that while IPA-3 alone did not impact the stalled replication fork, the combination of olaparib and IPA-3 significantly slowed the recovery of these forks compared with olaparib alone. In addition, combination of olaparib with IPA-3 significantly decreased the chromatin loading of RPA1 and RPA2 proteins compared with olaparib group (Fig. 5C). The combination of olaparib and IPA-3 led to reduced accumulation of RPA1 and RPA2 proteins on replication forks compared with treatment with olaparib alone (Fig. 5D). Overall, these findings showed that PAK1 activation plays a crucial role in managing the DNA replication stress response induced by olaparib.

PARP inhibitors promote cytotoxic effects by progressively inducing DSBs.^42^ We thus next speculated that IPA-3 might also enhance olaparib-mediated induction of DSBs. As shown in Figure 5E and F, combined treatment of ovarian cells with IPA-3 and olaparib significantly increased γ-H2AX levels compared with treatment with olaparib alone. Furthermore, RNA sequencing was performed to investigate how IPA-3, olaparib, and their combination influenced the activation of pathways associated with DSB repair. Figure 5G and H showed that the combined treatment significantly altered gene expression related to DNA damage regulation and repair. GSEA showed a significant up-regulation of the apoptosis pathway (Fig. 5I) and the P53 pathway (Fig. 5J). The heatmap and quantitative PCR results showed a significant up-regulation of the expression of DNA damage-related genes (Fig. 5K and L). These data suggest that IPA-3 may increase the susceptibility of OC cells to olaparib by inducing additional DNA damage.

### Olaparib and IPA-3 combination therapy synergistically suppresses OC xenograft tumor growth

**Figure 6:**
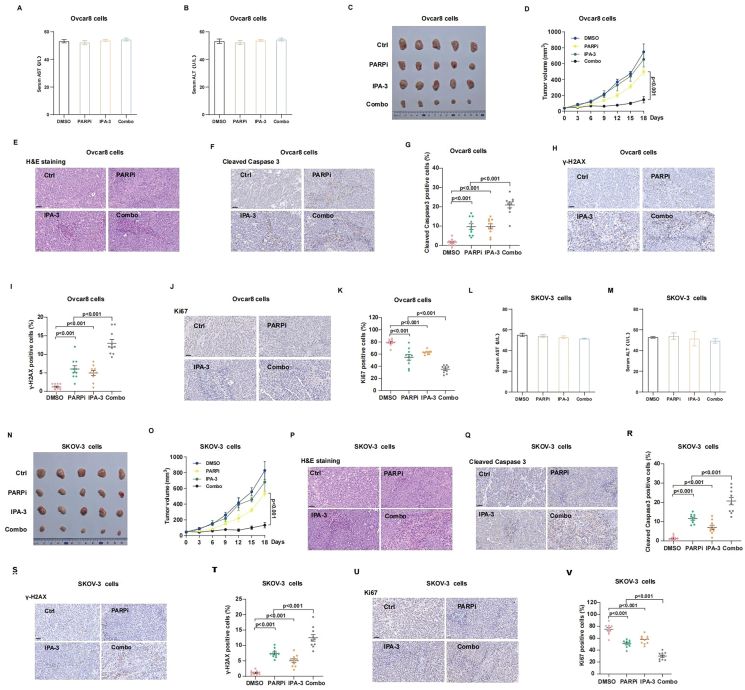
**Combination of IPA-3 and olaparib synergistically suppresses ovarian cancer xenograft tumor growth. OVCAR8 and SKOV-3 cells were subcutaneously implanted into NOD-SCID mice, and the animals were treated with control (DMSO), IPA-3 (10 mg/kg), olaparib (50 mg/kg), or their combination (intraperitoneally, 3 days × 6 times). (A, B, L, M)** Serum AST and ALT were measured for Ovcar8 (A, B) and SKOV-3 (L, M) xenografts. **(C, D, N, O)** Tumor images and growth curves for Ovcar8 (C, D) and SKOV-3 (N, O) xenografts. Data were expressed as mean ± standard error of the mean from five independent samples. Statistical significance was assessed by a two-tailed unpaired *t*-test. **(E–K, P–V)** Hematoxylin-eosin, Ki-67, γ-H2AX, and cleaved caspase-3 staining in tumor tissues, evaluated by immunohistochemistry for Ovcar8 (E–K) and SKOV-3 (P–V) xenografts. Quantification is shown in the corresponding panels. Images of 10 random fields per section were analyzed using ImageJ software. Scale bars = 50 μm. Statistical analysis was performed using a two-tailed *t*-test and two-way ANOVA. *P*-values < 0.05 were considered significant.

The xenograft mouse model was next used to examine the synergistic cytotoxicity effect of olaparib and IPA-3 combination therapy *in vivo*. To investigate whether the PAK1 inhibitor IPA-3 caused damage to the liver, we detected the changes in the serum AST and ALT levels of mice after IPA-3 treatment. The results showed that IPA-3 did not affect the levels of serum AST (Fig. 6A–L) and ALT (Fig. 6B–M) in mice. As depicted in Figure 6C, D, N, O, the combination of olaparib and IPA-3 markedly reduced tumor growth in Ovcar8 and SKOV-3 xenograft mouse models, in contrast to those in the single-agent groups. Immunohistochemistry analysis revealed that the combination of olaparib and IPA-3 significantly decreased Ki-67 expression and increased γ-H2AX and cleaved caspase-3 levels in the tumor compared with the single-agent treatments (Fig. 6E–K, P–V). Together, these results indicate the synergistic inhibitory effects of olaparib and IPA-3 on ovarian tumor growth through regulating DNA repair and cell apoptosis.

### IPA-3 enhances olaparib sensitivity in primary PDO and PDX models

**Figure 7:**
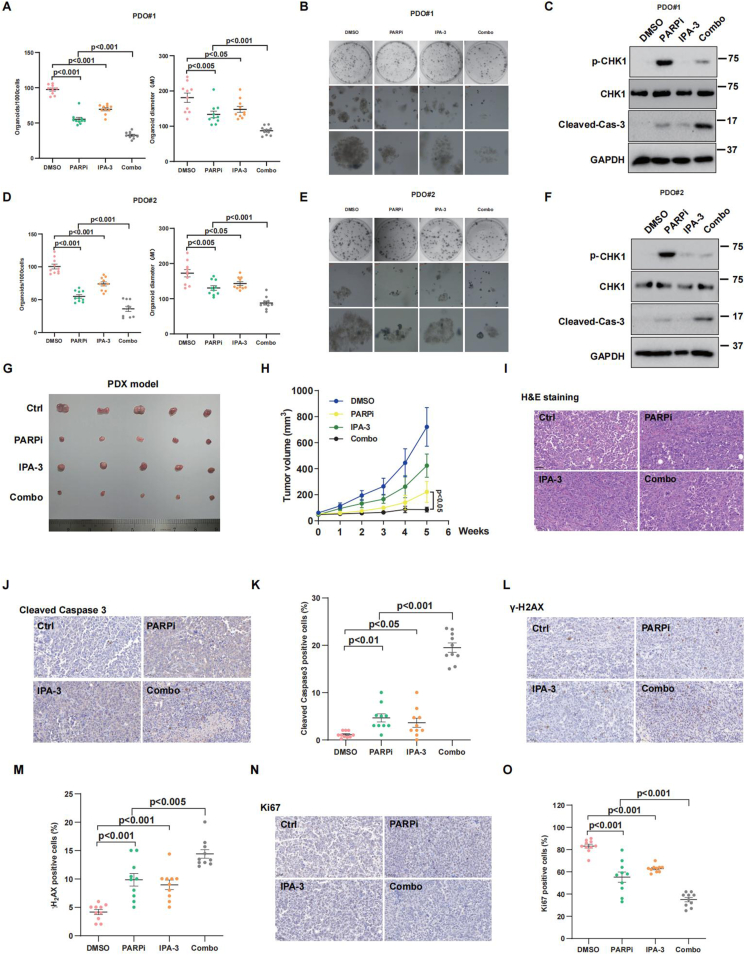
**Combination of IPA-3 and olaparib synergistically suppresses ovarian cancer cells' growth in patient-derived organoid and patient-derived xenograft models.(A, B, D, E)** Ovarian cancer organoids were treated with IPA-3 (200 nM), olaparib (200 nM), or both for 3 days. Representative bright-field images and quantitative analysis are shown. **(C, F)** Western blotting analysis of CHK1 phosphorylation and cleaved caspase-3 in ovarian cancer organoids treated with IPA-3 (200 nM), olaparib (200 nM), or both for 3 days. **(G, H)** Patient-derived xenograft models were established by transplanting tumor tissues into 6-week-old female BALB/c nude mice. Mice were treated with DMSO, IPA-3 (10 mg/kg), olaparib (50 mg/kg), or their combination. Tumor images (G) and growth curves (H) are shown. **(I–O)** Immunohistochemical analysis of hematoxylin-eosin, cleaved caspase-3, γ-H2AX, and Ki-67 levels in tumor tissues. Quantification of staining is shown in (K), (M), and (O). Data were represented as mean ± standard deviation. Images of 10 random fields per section were recorded for analysis. Statistical significance was assessed using a two-tailed *t*-test and a two-way ANOVA. *P*-values < 0.05 were considered significant. Scale bars = 50 μm.

Two OC patient-derived tumor organoids, namely #1 and #2, were employed to conduct a more in-depth assessment of the sensitivity regarding the combined treatment of olaparib and IPA-3. As shown in Figure 7A, B, D, E, the reduced formation and smaller diameter of the two organoids indicated that the combination of the two agents exhibited synergistic cytotoxicity in the *ex vivo* model. In addition, olaparib-induced caspase-3 cleavage was increased when combined with IPA-3 in the two PDO models (Fig. 7C–F), suggesting that the combination of olaparib and IPA-3 has a far greater effect on organoid apoptosis compared with the single-agent groups.

We evaluated the effectiveness of olaparib, both as a monotherapy and in conjunction with IPA-3, using the PDX model. As shown in Figure 7G and H, olaparib combined with IPA-3 resulted in additional suppression of tumor growth in the PDX model. In addition, immunohistochemistry data showed that olaparib-reduced Ki-67 expression, as well as increased γ-H2AX and cleaved caspase-3, was aggravated when added with IPA-3 (Fig. 7I–O). Collectively, these findings indicate that combining IPA-3 with olaparib effectively enhances the sensitivity of ovarian tumors to olaparib.

## Discussion

PARP inhibitors are authorized for maintenance therapy in high-grade OC, particularly for patients with HR repair deficiency.^37^^,^^43^^,^^44^ Nonetheless, many OC patients remain unresponsive to PARP inhibitors or develop adaptive resistance over time.^45^^,^^46^ Therefore, exploring agents that target HR repair pathways to sensitize ovarian tumor cells to or bypass resistance to PARP inhibitors is a promising approach to overcome therapeutic challenges in OC. This study found that serine/threonine kinase PAK1 regulated HR repair, and its knockdown enhanced olaparib-induced cytotoxicity. Moreover, the combination of a PAK1 inhibitor with olaparib synergistically increased olaparib's efficacy in both *in vitro* and *in vivo* studies. Our study elucidated the role and molecular mechanisms of PAK1 in enhancing PARP inhibitor sensitivity in OC cells, suggesting potential therapeutic applications.

Other members of the PAK family, such as PAK2, are also associated with a variety of malignant tumors.^47^ PAK2 is significantly amplified and phosphorylated in gastric cancer. Among them, cyclin-dependent kinase 12 (CDK12) can phosphorylate PAK2 to promote the progression of gastric cancer. PAK2 has the potential to be used as a serum diagnostic marker for pancreatic cancer. PAK2 is activated after being cleaved by caspase and subsequently induces early apoptosis. Mutations in the PAK3 gene lead to changes in its function, affecting the cell's sensitivity to DNA damage.^48^ However, the exact relationship between PAK2/PAK3 and cancer still remains to be fully elucidated, and further research is needed.

PARP inhibitors can cause unrepaired SSBs to accumulate or obstruct replisome progression, leading to DSBs that necessitate HR for repair.^49^^,^^50^ In addition, HR repair genes, including BRCA1/2, RAD51, RPA2, and CHK1, are well-known to play a direct role in the protection, restart, and restoration of replication forks, which contribute to the maintenance of genomic stability during DNA replication.^51^, ^52^, ^53^ Our previous results demonstrate that PAK1 directly interacts with and phosphorylates RPA1 to engage in replication stress-induced recruitment of the RPA protein and DNA damage response.^32^ However, the specific role of PAK1 in the HR repair pathway remains to be explored. This study demonstrated that PAK1 depletion or inhibition reduced HR repair efficiency and increased the sensitivity of OC cells to olaparib. In addition, we revealed that olaparib-induced replication stress and DSB damage were all attenuated when cells were treated with PAK1 inhibitor IPA-3, indicating that PAK1-mediated cytotoxicity induced by olaparib treatment is also through HR repair. Given the essential function of the RPA complex in facilitating HR repair during replication stress and DSB damage, it is expected that PAK1 influences HR repair both directly and indirectly through RPA.^54^, ^55^, ^56^ Indeed, our results found that olaparib-induced RPA protein recruitment on stalled replication fork also depended on the presence of PAK1. However, given the extensive range of RPA-recruited HR regulatory proteins, the detailed molecular mechanism of how the PAK1-RPA pathway regulates HR repair in response to olaparib-induced cytotoxicity is still obscure. Moreover, besides RPA, the additional targets of PAK1 contributing to the enhanced sensitivity to olaparib also need to be further identified in subsequent studies to comprehensively elucidate the role of PAK1 in HR repair.

In light of bioinformatics analysis and prior studies, approximately 25% of OC patients exhibit amplification of PAK1 within the chromosomal region 11q13.^57^^,^^58^ PAK1 expression is inversely related to overall survival in OC, suggesting its potential as a biomarker for predicting chemotherapy outcomes in this disease.^59^^,^^60^ Targeting PAK1, alone or in combination with other agents, presents a promising strategy for treating OC patients. Our results showed that IPA-3 alone had only a modest effect on the survival of OC cells through colony formation assays. However, the combination of IPA-3 and olaparib synergistically enhanced olaparib-induced cytotoxicity *in vitro* and in CDX models. We employed PDO and PDX models, which replicate characteristics of natural tumors, to further investigate PAK1's influence on the therapeutic response to PARP inhibitors in OC patients. The study demonstrated that organoids and PDXs displayed phenotypes similar to cell lines and CDX models. Inhibition of PAK1 with IPA-3 enhanced tumor sensitivity to olaparib, indicating the potential for clinical application of IPA-3 combined with olaparib in treating OC patients. Meanwhile, although it was reported that high doses of PAK1 inhibitor alone could suppress the growth of certain cancer cells, the unacceptable side effects of PAK1 inhibitors on other organs pose a challenge.^61^ Combining PAK1 with PARP inhibitors offers a promising clinical strategy to enhance therapeutic efficacy and reduce potential side effects. The current evidence supports the incorporation of IPA-3 into combination therapies to overcome drug resistance and improve therapeutic efficacy across various cancers.^62^, ^63^, ^64^, ^65^ Our findings corroborate and expand upon these insights, revealing that IPA-3-based combinations significantly enhance olaparib sensitivity in OC patients.

IPA-3 is a potent PAK1 inhibitor that binds to the kinase's autoinhibitory domain, blocking activation through a non-ATP-competitive mechanism.^66^ This distinctive mode of action confers greater selectivity than ATP-competitive inhibitors, effectively inhibiting PAK1-mediated oncogenic signaling.^63^ Preclinical studies across multiple cancer models, including acute myeloid leukemia, prostate cancer, esophageal cancer, and pancreatic cancer, demonstrate IPA-3's ability to suppress tumor cell proliferation and metastasis.^67^, ^68^, ^69^, ^70^ Additionally, its concurrent inhibition of VEGF-mediated signaling further confers anti-angiogenic effects, collectively highlighting broad therapeutic potential.^71^ Despite preclinical validation of IPA-3, clinical development of PAK1 inhibitors remains challenging. PF-3758309, an ATP-competitive PAK1 inhibitor, demonstrates potent anti-tumor activity across preclinical models by inducing apoptosis and suppressing proliferation.^72^^,^^73^ Despite this, its clinical translation failed in phase I trials due to suboptimal pharmacokinetics, particularly poor oral bioavailability.^74^ In contrast, BJG-05-039, a PROTAC-based PAK1 degrader, represents a paradigm shift by coupling kinase inhibition with ubiquitin-mediated protein degradation, achieving enhanced target specificity.^75^ Nevertheless, no PAK1-targeted agent has advanced to phase III trials, with persistent challenges including limited bioavailability, off-target effects, and dose-limiting toxicities.^74^ Our data demonstrate that a synergistic combination of IPA-3 with PARP inhibitors enables dose reduction while maintaining efficacy, a strategy that may alleviate toxicity barriers. Clinical translation efforts should prioritize biomarker-driven enrollment in PAK1-amplified populations to optimize therapeutic index.

In summary, the research demonstrated that PAK1 controlled HR repair and its inhibition worked together to increase the cytotoxic effects of olaparib in OC cells, suggesting a potential approach to improve the efficacy of therapies based on PARP inhibitors. Our study provides new perspectives on the molecular mechanisms that influence PARP inhibitor sensitivity in OC, suggesting potential for future PAK1/PARP inhibitor combination clinical trials to benefit OC patients.

## CRediT authorship contribution statement

**Changying Li:** Formal analysis, Visualization, Writing – original draft, Funding acquisition, Data curation. **Xinyan Li:** Validation, Data curation, Formal analysis. **Ming Gao:** Writing – review & editing. **Ye-Xiong Li:** Writing – review & editing, Project administration, Supervision. **Zhenkun Lou:** Writing – review & editing, Project administration, Validation, Methodology.

## Funding

This work was supported by grants from the 10.13039/501100001809National Natural Science Foundation of China (No. 82403784), 10.13039/501100002858China Postdoctoral Science Foundation (No. 2022TQ0043, 2023M730331), and the 10.13039/501100012226Fundamental Research Funds for the Central Universities, Peking Union Medical College (No. 2023-CICAMS-3332023029).

## Conflict of interests

The authors declared no competing interests.
